# CuLi_2_Sn and Cu_2_LiSn: Characterization by single crystal XRD and structural discussion towards new anode materials for Li-ion batteries

**DOI:** 10.1016/j.jssc.2014.08.006

**Published:** 2014-12

**Authors:** Siegfried Fürtauer, Herta S. Effenberger, Hans Flandorfer

**Affiliations:** a Institute of Inorganic Chemistry (Materials Chemistry), University of Vienna, Währingerstraße 42, A-1090 Wien, Vienna; b Institute of Mineralogy and Crystallography, University of Vienna, Althanstraße 14, A-1090 Wien, Vienna

**Keywords:** Cu–Li–Sn, Lithium, Stannide, Single-crystal XRD, Crystal structure, Li-ion battery

## Abstract

The stannides CuLi_2_Sn (CSD-427095) and Cu_2_LiSn (CSD-427096) were synthesized by induction melting of the pure elements and annealing at 400 °C. The phases were reinvestigated by X-ray powder and single-crystal X-ray diffractometry. Within both crystal structures the ordered CuSn and Cu_2_Sn lattices form channels which host Cu and Li atoms at partly mixed occupied positions exhibiting extensive vacancies. For CuLi_2_Sn, the space group F-43m. was verified (structure type CuHg_2_Ti; *a*=6.295(2) Å; *wR*_2_(*F*²)=0.0355 for 78 unique reflections). The 4(*c*) and 4(*d*) positions are occupied by Cu atoms and Cu+Li atoms, respectively. For Cu_2_LiSn, the space group *P*6_3_/*mmc* was confirmed (structure type InPt_2_Gd; *a*=4.3022(15) Å, *c*=7.618(3) Å; *wR*_2_(*F*²)=0.060 for 199 unique reflections). The Cu and Li atoms exhibit extensive disorder; they are distributed over the partly occupied positions 2(*a*), 2(*b*) and 4(*e*). Both phases seem to be interesting in terms of application of Cu–Sn alloys as anode materials for Li-ion batteries.

## Highlights

• First single crystal investigation of CuLi_2_Sn and Cu_2_LiSn clarifies contradictions from literature.
• Lithium atoms are ordered in channels, which is interesting for application as anode materials for lithium ion batteries.
• Structural relationships to binary Cu–Sn-phases are shown.
• Close structural relationship between both ternary phases exists.

## graphical abstract

**Figure:**
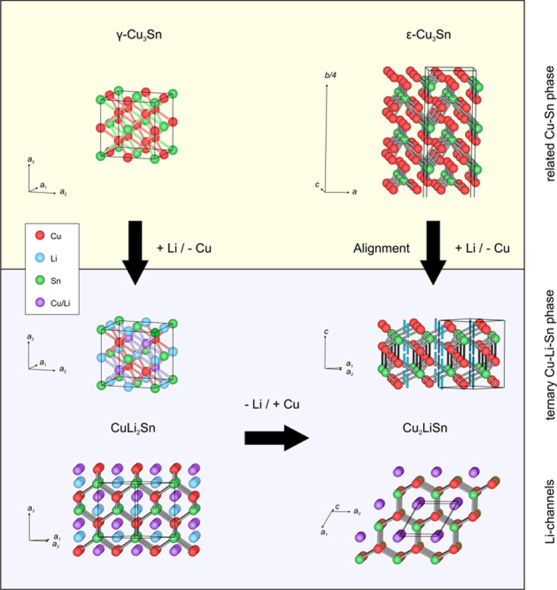

## 1 Introduction

Intermetallic compounds in Li-containing systems are in focus of research due to the promising application of intermetallic alloy electrodes in enhanced Li-ion batteries [1], [2], [3]. Special interest has been drawn to Sn containing systems. The binary compound Li_17_Sn_4_ offers, for example, a theoretical energy density of 960 mAh/g, compared to a theoretical energy density of 372 mAh/g for LiC_6_, which is formed in currently commercially used graphite anodes [4]. The pronounced volume change by reversible insertion of Li atoms into the Sn containing compound, however, is a main obstacle for practical application of such alloys at room temperature. Cu–Li–Sn compounds are a promising prospective material system for improved anode materials. Cu probably acts as a buffer to overcome the mechanical stress and electrode destruction during cycling. Based on electrochemical insertion experiments, various authors [5], [6], [7], [8] proposed the reaction mechanisms

Cu_6_Sn_5_+10Li→5CuLi_2_Sn+Cu

5CuLi_2_Sn+11.25Li→5Li_4.25_Sn+5Cu.

According to this mechanisms the insertion of Li atoms into the compound Cu_6_Sn_5_ [9] (related to the structure type NiAs) results – besides the formation of a solid solution between Sn and Li in Cu – in the formation of the ternary intermetallic phase CuLi_2_Sn. Continuing the insertion of Li atoms into CuLi_2_Sn, again Cu precipitates and the binary compound Li_4.25_Sn (=Li_17_Sn_4_ [10]), exhibiting the highest known Li content, forms. Despite such electrochemical studies there is still scarce knowledge about the fundamentals of the ternary system Cu–Li–Sn with respect to phase relations, crystallographic and thermochemical data [11]. In contrast, the binary systems Cu−Sn [12], [13], Cu−Li [14] and Li−Sn [15], [16] and their constituting phases have been extensively investigated. For tentative applications of the Cu–Li–Sn system in competitive battery systems, phase relations and knowledge of ternary intermetallic compounds is mandatory. Though the reliable prediction of the formation of ternary compounds based on calculations is limited to some extent, complementary experimental studies are indispensable.

The ternary compound CuLi_2_Sn was first synthesized by Pauly et al. [17] in the 1960s; they found the structure type CuHg_2_Ti for this phase based on X-ray powder diffraction data, a *fcc* atomic arrangement exhibiting the acentric space-group symmetry F-43m. Later on, Schuster et al. [18], [19] refined this crystal structure again with powder X-ray techniques, but they found the centric space group Fm-3m. The two structure models are essentially the same but differ with respect to an order–disorder phenomenon. In the acentric structure model Li and Cu are ordered to a large extent occupying the 4(*c*) and 4(*d*) position whereas in the centric structure model these two atoms are allocated randomly forming the 8(*c*) mixed occupied site (see Table 1).

**Table 1:** Literature crystal structures for CuLi_2_Sn and Cu_2_LiSn.

**Phase**	**Isotype**	**Pearson symbol**	**Space group**	**SG no.**	***a*****(Å)**	***c*****(Å)**	**Atom**	**Site**	***x***	***y***	***z***	**Occ.**	**Ref.**
**CuLi**_**2**_**Sn**	CuHg_2_Ti	cF16	F-43m.	216	6.282(3)	–	Sn	4*a*	0	0	0	1.0	[17]
							Li1	4*b*	0.5	0.5	0.5	1.0	
							Li2	4*c*	0.25	0.25	0.25	1.0	
							Cu	4*d*	0.75	0.75	0.75	1.0	

**CuLi**_**2**_**Sn**	AlCu_2_Mn	cF16	Fm-3m.	225	6.263	–	Sn	4*a*	0	0	0	1.0	[18], [19]
							Li1	4*b*	0.5	0.5	0.5	1.0	
							Li2	8*c*	0.25	0.25	0.25	0.5	
							Cu	8*c*	0.25	0.25	0.25	0.5	

**Cu**_**2**_**LiSn**	InPt_2_Gd	hP8	*P*6_3_/*mmc*	194	4.303	7.637	Li	2*a*	0	0	0	1.0	[20]
							Sn	2*c*	1/3	2/3	0.25	1.0	
							Cu	4*f*	1/3	2/3	0.583	1.0	

The ternary compound Cu_2_LiSn was only described by Kripyakevich et al. [20]. They found hexagonal symmetry (space group *P*6_3_/*mmc*) from powder-diffraction data (see also Table 1).

Even the structure types could be characterized by these authors, details about mixed and/or partly occupied sites as well as the chemical variability and the formation of solid solutions maintained undetected in those days. The actual work aims at an improved and detailed description of the crystal structures of both phases by single-crystal X-ray diffraction and a clarification of the contradicting literature data. Furthermore, the possibility of a mutual substitution of Li and Cu atoms was of interest for the Li insertion mechanisms required for batteries. Although the resulting nomenclature of both compounds, considering occupation ratios of Cu and Li atoms, would be Cu_1+*x*_Li_2−*x*_Sn for CuLi_2_Sn and Cu_2+*x*_Li_1−*y*_Sn for Cu_2_LiSn, the designations with integer values are preferred for easier readability throughout this paper.

## 2 Materials and methods

### 2.1 Sample preparation

As inserts for sample preparations served the pure elements Cu (99.98 at%, wire, Goodfellow, Cambridge, UK), Li (99.8 at%, wire, Alfa Aesar, Karlsruhe, Germany) and Sn (99.95 at%, ingot, Advent, Oxford, UK). The Cu wire was treated in a H_2_-flow for 5 h at 300 °C to remove the oxide layer at the surface. The Li wire was stored originally in mineral oil, which was removed by n-hexane in a supersonic bath followed by vacuum evaporation of the solvent. Visible oxidation spots occurring partially at the surface were removed mechanically with a scalpel. Inside a glove box under Ar atmosphere (<5 ppm O_2_/H_2_O), the metal pieces were assembled in tantalum crucibles, which were made by deep-drawing of a 0.4 mm tantalum sheet. For welding the crucibles in argon atmosphere, an arc furnace with a tungsten electrode of 1.6 mm was used. During the welding process, the crucibles were chilled by a water cooled Cu mount. The filled crucibles were put into an induction furnace at 1100 °C for only 10–20 s to prevent high temperature fatigue of the welding seam. This procedure allowed melting of the input. Repetition of the melting process twice with turning the crucible upside down between the heating steps assured homogenous mixing. After that, the crucibles were sealed in quartz glass tubes under vacuum and annealed at 400 °C in a muffle furnace. After 35 days the samples were quenched in cold water and opened in the glove box with a bolt cutter. The obtained alloys were very brittle and had a coloured tint (CuLi_2_Sn: metallic pink; Cu_2_LiSn: metallic violet).

### 2.2 Powder XRD

In a glove box small amounts of the samples were grinded with a Durit^®^mortar. The obtained powder had a particle size <25 μm, therefore sieving was not necessary. For powder XRD measurements a Bragg–Brentano diffractometer (*Θ*/2*Θ*-geometry) with a Cu radiation source (40 kV/40 mA) and a Ni filter were used. Signals were detected by a strip detector. A silicon mono-crystal was used as sample holder. The powdered sample was fixed by petroleum jelly on the sample holder in the glove box. Oxidation was prevented by an X-ray amorphous cap of polycarbonate, which maintained the protection gas atmosphere on the sample. The obtained patterns were evaluated by Rietveld-refinements using Topas3^®^ [21] software.

### 2.3 Single-crystal XRD

The models of the atomic arrangement published so far [17], [18], [19] are based on X-ray investigations by Debye–Scherrer and Straumanis exposures in combination with powder-diffractometer measurements. These methods gave rough information about the structure type only. However, the tentative application for Li-ion batteries requires a detailed knowledge about possible substitution mechanisms which is available only by single-crystal XRD investigations. Samples again had to be protected from interactions with atmosphere. Small amounts of gritty bulk samples were fixed in the glove box with amorphous petroleum jelly between two object plates. Slightly pushing and sliding of the object plates crushed the bulk samples into smaller particles and covered it entirely with the grease. Crystal chips suitable for the X-ray investigations were selected outside the glove box under a binocular microscope (up to 400 fold magnification) and handled with fine acupuncture needles. The respective crystals were approximately 50 μm in diameter; they were fixed onto a glass capillary. During this procedure the samples were kept under petroleum jelly which protected them from altering due to interaction with moisture from the air. Single-crystal XRD was performed with a four-circle Nonius Kappa diffractometer with a CCD detector and a 300 μm capillary optics collimator (Mo*K*α radiation, graphite monochromator). Isothermal conditions at 290 K were enabled by a continuous stream of nitrogen enclosing the single crystal, which protected the sample from corrosion due to moisture in the atmosphere. After data collection, the unit cell parameters were obtained from least-square refinements of all observed 2*ϑ* values. Corrections for Lorentz, polarization and absorption effects (multi-scan method) were applied. Complex scattering functions from Wilson [22] and the programs “Collect” [23], [24], “SHELXL-97” [25], [26], [27] as well as “PLATON” [28] were used.

## 3 Results

**Fig. 1:**
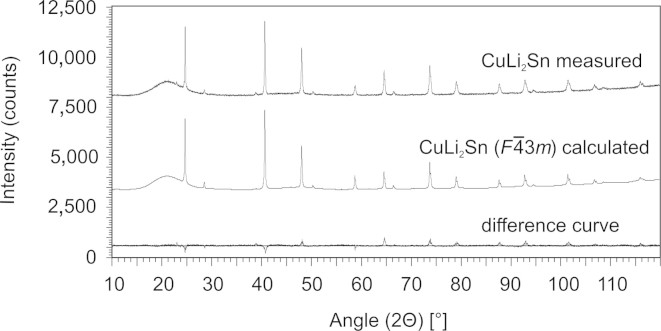
Powder diffractogram of CuLi_2_Sn: measured, calculated and difference patterns.

**Fig. 2:**
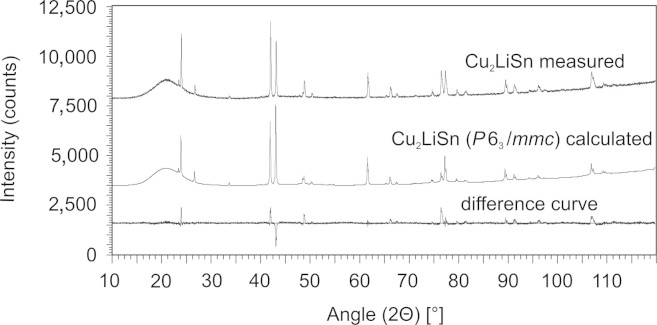
Powder diffractogram of Cu_2_LiSn: measured, calculated and difference patterns.

Powder XRD measurements were applied to check phase homogeneity of the synthesized samples. Observed diffractograms and refinement results are given in Fig. 1, Fig. 2, respectively. No impurities or any additional phases could be detected. The wide peak at low angles originates from the protection cap.

Details of the data collection of single-crystal X-ray investigations and structure refinements are compiled in Table 2, and fractional coordinates and interatomic bond distances are given in Table 3, Table 4, respectively. For both cases the atomic coordinates from the literature [17], [18], [19], [20] served in the starting set of the structure refinement. The space-group symmetries agreed with the extinction rules. Successive Fourier and difference Fourier summations revealed details of the atomic arrangements. The chemical compositions found from structure investigations are in accordance with the atomic fractions of the input for syntheses. Structural parameters including anisotropic displacements were refined (Table 3). In CuLi_2_Sn, the atomic sites were considered as fully but partly mixed occupied according to the chemical formula Cu_1+*x*_Li_2−*x*_Sn including occupation ratios (see also Table 3). The highest peaks in the electron densities found in the final difference Fourier maps are located close to Sn or Cu sites. In Cu_2_LiSn occurs an extensive atomic dislocation going along with partial occupied sites (see Section 4).

**Table 2:** Single-crystal X-ray data-collection and crystal structure refinements of CuLi_2_Sn and Cu_2_LiSn. Cell parameters determined from PXRD are given in square brackets.

(Ideal) Chemical formula	CuLi_2_Sn	Cu_2_LiSn
Nominal composition of sample preparation	Cu_0.25_Li_0.50_Sn_0.25_	Cu_0.50_Li_0.25_Sn_0.25_
Chemical formula including occupation ratios	Cu_1+*x*_Li_2−*x*_Sn	Cu_2+*x*_Li_1−*y*_Sn
*a* (Å)	6.295(2)	4.3022(15)
	[6.27922(8)]	[4.30711(8)]
*c* (Å)	—	7.618(3)
		[7.6198(1)]
Space group (no.)	F-43m. (216)	*P*6_3_*/mmc* (194)
*V* (Å^³^)	249.5	122.1
Pearson symbol	cF16	hP8
*Z*	4	2
*ρ*_*calc*_ (g cm^-3^)/*μ*(Mo*K*α) (mm^−1^)	5.22/18.1	6.87/27.0
Crystal dimensions (μm)	75×95×120	35×60×60
Range of data collection (±*h*±*k*±*l*) (°)	3<2ϑ<70	3<2ϑ<70
Number of images/rotation angle per image (°)	431/2.0	603/ .0
Scan mode (*φ*-scans at distinct *ω*-angles)	9 *φ*-scans	10 *φ*-scans
Scan time (s/°)/frame size 621×576 pixels (binned mode)	20	140
Detector-to-sample distance (mm)	30	30
Measured reflections	1018	2015
Unique reflections (*n*)/observed reflections (*F*_*o*_>4*σ*(*F*_*o*_))	78/78	199/125
*R*_*int*_=Σ∣*F*_*o*_^2^−*F*_*o*_^2^(mean)∣/Σ*F*_*o*_^2^	0.035	0.034
Extinction parameter *k*:		
*F*_*c*_^⁎^=*F*_*c*_*k*(1+0.001*F*_*c*_^2^λ^3^/sin(2*ϑ*))^–1/4^	0.0086(16)	0.022(5)
*R*_1_=Σ(∣∣*F*_*o*_∣-∣*F*_*c*_∣∣)/Σ*F*_*o*_ (all/observed reflections)	0.013/0.013	0.020/0.022
*wR*_2_=(Σ*w*(*F*_*o*_^2^-*F*_*c*_^2^)^2^/Σ∣*wF*_*o*_^4^)^1/2^	0.0355	0.060
GooF={Σ(*w*(*F*_*o*_^2^−*F*_*c*_^2^)^2^)/(*n−p*)}^0.5^	1.32	1.03
Max *Δ*/*σ*; Number of variable parameters (*p*)	<0.001; 9	<0.001; 14
Final difference Fourier map (eÅ^−3^)	−0.35 to +0.57	−1.26 to +1.01
Parameters *a*/*b* for weighting scheme	0.022/0.19	0.039/0.50
Racemic twin component	0.09(9)	—
Volume per atom (Å^³^)	15.6	15.3

**Table 3:** Fractional atomic coordinates and displacement parameters for CuLi_2_Sn and Cu_2_LiSn. The anisotropic displacement parameters are defined as: exp(−2*π*^2^Σ^3^_*i*=1_Σ^3^_*i*=1_*U*_*ij*_*a*^⁎^_*i*_*a*^⁎^_*j*_*h*_*i*_*h*_*j*_).

	Occupation	Wyckoff letter	Site symmetry	*x*	*y*	*z*	*U*_*equiv*_*U*_*iso*_	*U*_11_	*U*_22_	*U*_33_	*U*_23_	*U*_13_	*U*_12_
**CuLi**_**2**_**Sn**													
Sn	Sn_1.00_	4(*a*)	−43*m*	0	0	0	0.0178(3)	0.0178(3)	=*U*_11_	=*U*_11_	0	0	0
Cu	Cu_1.00_	4(*c*)	−43*m*	1/4	1/4	1/4	0.0189(12)	0.0189(12)	=*U*_11_	=*U*_11_	0	0	0
Li	Li_1.00_	4(*b*)	−43*m*	0	0	1/2	0.016(7)	0.016(7)	=*U*_11_	=*U*_11_	0	0	0
Li/Cu	Li_0.846(15)_Cu_0.154_	4(*d*)	−43*m*	3/4	3/4	3/4	0.034(9)	0.034(9)	=*U*_11_	=*U*_11_	0	0	0

**Cu**_**2**_**LiSn**													
Sn	Sn_1.00_	2(*c*)	−6*m*2	1/3	2/3	1/4	0.0174(3)	0.0180(3)	=*U*_11_	0.0161(3)	0	0	=(1/2)⁎*U*_11_
Cu	Cu_1.00_	4(*f*)	3*m*	1/3	2/3	0.58905(9)	0.0191(3)	0.0200(3)	=*U*_11_	0.0172(4)	0	0	=(1/2)⁎*U*_11_
Li/Cu1	Li_0.8(6)_^a^	2(*a*)	−3*m*	0	0	0	0.028(6)^b^	0.019(5)^b^	=*U*_11_	0.045(15)^b^	0	0	=(1/2)⁎*U*_11_
Li/Cu2	Li_0.5(6)_^a^	4(*e*)	3*m*	0	0	0.090(5)	0.028(6)^b^	0.019(5)^b^	=*U*_11_	0.045(15)^b^	0	0	=(1/2)⁎*U*_11_
Li/Cu3	Li_0.6(6)_^a^	2(*b*)	−6*m*2	0	0	¼	0.021(9)						

a Excess of Li-atoms was allowed due to electron density.

b The displacement parameters for the atoms Li/Cu1 and Li/Cu2 in Cu_2_LiSn were constrained.

**Table 4:** Interatomic bond lengths (Å) of CuLi_2_Sn and Cu_2_LiSn.

**CuLi**_**2**_**Sn**			
Sn–Cu^*0, iii, viii, xiii*^	2.7258(10)	Li–Li/Cu^*xiii, xvi, xvii, xviii*^	2.7258(10)
Sn–Li/Cu^*xvii, xviii, xix, xx*^	2.7258(10)	Li–Cu^*0, v, x xiii*^	2.7258(10)

Cu–Li^*0, iv, ix, xiv*^	2.7258(10)	Li/Cu–Li^*i, vii, xii, xiv*^	2.7258(10)
Cu–Sn^*0, vi, xi, xiv*^	2.7258(10)	Li/Cu–Sn^*ii, vii, xii, xv*^	2.7258(10)
Symmetry code: not specified and ^*0*^*x*,*y*,*z*; ^*i*^*x*+1, *y*+1, *z*; ^*ii*^*x*+1, *y*+1, *z*+1; ^*iii*^*x*, *y*−1/2, *z*−1/2; ^*iv*^*x*, *y*+1/2, *z*−1/2; ^*v*^*x*, *y*−1/2, *z*+1/2; ^*vi*^*x*, *y*+1/2, *z*+1/2; ^*vii*^*x*+1, *y*+1/2, *z*+1/2; ^*viii*^*x*−1/2, *y*, *z*−1/2; ^*ix*^*x*+1/2, *y*, *z*−1/2; ^*x*^*x*−1/2, *y*, *z*+1/2; ^*xi*^*x*+1/2, *y*, *z*+1/2; ^*xii*^*x*+1/2, *y*+1, *z*+1/2; ^*xiii*^*x*−1/2, *y*−1/2, *z*; ^*xiv*^*x*+1/2, *y*+1/2, *z*; ^*xv*^*x*+1/2, *y*+1/2, *z*+1; ^*xvi*^*x*−1, *y*−1, *z*; ^*xvii*^*x*−1, *y*−1/2, *z*−1/2; ^*xviii*^*x*−1/2, *y*−1, *z*−1/2; ^*xix*^*x*−1/2, *y*−1/2, *z*−1; ^*xx*^*x*−1, *y*−1, *z*−1			

**Cu**_**2**_**LiSn**			
Sn–Li3^*0, iii, iv*^	2.4839(9)	Li2···Li1^0^	0.69(4)
Sn–Cu^*0, xiv*^	2.5829(12)	Li2···Li3^*0*^	1.22(4)
Sn–Cu^*ix, x, xi, xviii, xix, xx*^	2.7700(9)	Li2···Li2^*v*^	1.37(8)
Sn–Li2^*0, iii, xiv, xv*^	2.767(17)	Li2–Li2^*xiii*^	2.44(8)
		Li2–Cu^*xvii, xviii, xix*^	2.4839(9)
Cu–Cu^*xvi*^	2.4523(17)	Li2–Li3^*v*^	2.59(4)
Cu–Li2^*xxi, xxii, xxiii*^	2.4839(9)	Li2–Sn^*0, i, ii*^	2.77(2)
Cu–Li1^*xiii, xiv, xv*^	2.5748(9)	Li2–Cu^*xii, xiii, xiv*^	2.83(2)
Cu–Sn^*0*^	2.5829(12)		
Cu–Sn^*ix, x, xi*^	2.7700(9)	Li3···Li2^*0, xiii*^	1.22(0.04)
Cu–Li3^*viii, ix, x*^	2.7700(9)	Li3···Li1^*0, xiii*^	1.9045(7)
Cu–Li2^*xiv*^	2.834(18)	Li3–Sn^*0, i, ii*^	2.4839(9)
		Li3–Li2^*v, xxi*^	2.59(4)
Li1···Li2^*0, v*^	0.69(4)	Li3–Cu^*viii, ix, x, xvii, xviii, xix*^	2.7700(9)
Li1···Li3^*0, v*^	1.9045(7)		
Li1–Cu^*xii, xiii, xiv, xvii, xviii, xix*^	2.5748(9)		
Symmetry code: not specified and ^*0*^*x*,*y*,*z*; ^*i*^*x*−1, *y*−1, *z*; ^*ii*^*x*, *y*−1, *z*; ^*iii*^*x*, *y*+1, *z*; ^*iv*^*x*+1, *y*+1, *z*; ^*v*^ −*x*, −*y*, −*z*; ^*vi*^ −*x*, −*y*+1, −*z*; ^*vii*^ −*x*+1, −*y*+1, −*z*; ^*viii*^ −*x*, −*y*, −*z*+1; ^*ix*^ −*x*, −*y*+1, −*z*+1; ^*x*^ −*x*+1, −*y*+1, −*z*+1; ^*xi*^ −*x*+1, −*y*+2, −*z*+1; ^*xii*^*x*−1, *x*−*y*, −*z*+1/2; ^*xiii*^*x*, *x*−*y*, −*z*+1/2; ^*xiv*^*x*, *x*−*y*+1, −*z*+1/2; ^*xv*^*x*+1, *x*−*y*+1, −*z*+1/2; ^*xvi*^*x*, *x*−*y*+1, −*z*+3/2; ^*xvii*^ −*x*, −*x*+*y*−1, *z*−1/2; ^*xviii*^ −*x*, −*x*+*y*, *z*−1/2; ^*xix*^ −*x*+1, −*x*+*y*, *z*−1/2; ^*xx*^ −*x*+1, −*x*+*y*+1, *z*−1/2; ^*xxi*^ −*x*, −*x*+*y*, *z*+1/2; ^*xxii*^ −*x*, −*x*+*y*+1, *z*+1/2; ^*xxiii*^ −*x*+1, −*x*+*y*+1, *z*+1/2			

## 4 Discussion

### 4.1 CuLi_2_Sn

All atoms occupy special positions. Besides changed point symmetries in the two proposed space groups Fm-3m. and F-43m. the sites 4(*a*) and 4(*b*) are identical and occupied by Sn and Li atoms, respectively, corresponding to the NaCl structure type. All cubic eightfold coordinated voids are occupied by further Li atoms and by the Cu atoms. The acentric space group F-43m allows order between the sites 4(*c*) (1/4 1/4 1/4) and 4(*d*) (3/4 3/4 3/4), whereas in *Fm*3¯*m* these two sites are crystallographically identical – site 8(*c*) – resulting in a statistical occupation by Cu and Li atoms. Different syntheses methods are quoted as tentative reasons for the distinct distribution of the Li and Cu atoms. Already Schuster et al. [19] discussed these differences and mentioned that some of the intensities in the powder pattern vary significantly. Actual refinements were performed for both structure models. As an example, the reflections 111, 200, 220 and 311 have structure-factor ratios of 52:32:100:84 in the centrosymmetric and 72:32:100:66 in the acentric space group if the ideal composition CuLi_2_Sn is considered [29]; single-crystal data clearly correspond better with the latter one: 72:20:100:54.

**Fig. 3:**
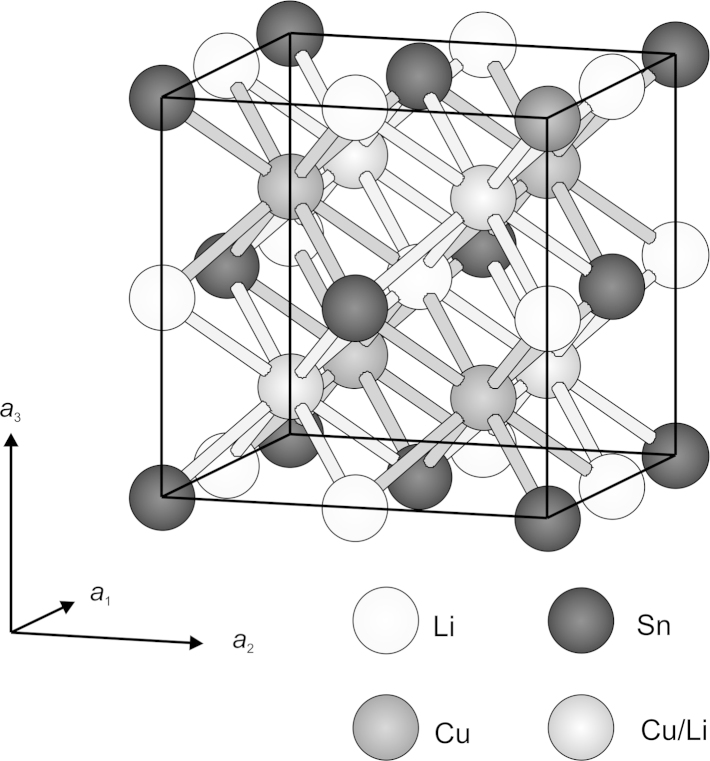
Unit cell of CuLi_2_Sn phase; Sn atoms at 4(*a*)-site, Li atoms at 4(*b*)-site, Cu atoms at 4(*c*)-site, and mixed occupation of Cu/Li atoms at 4(*d*)-site.

After a few cycles of the least-squares refinement in the acentric space group F-43m a satisfactory result could be observed. Especially the difference Fourier summation calculated with the three sites 4(*a*)-Sn, 4(*c*)- and 4(*d*)-(Cu,Li) clearly showed the highest residual density at the site 4(*b*) which could be refined considering full occupation by Li atoms successfully (see Table 3 and Fig. 3). In contrast, refinements in space group *Fm*3¯*m* with the Sn atoms at the site 4(*a*) and (Cu,Li) at 8(*c*) astonishingly did not show further residual densities at the site 4(*b*). Trials to refine this latter position occupied by Li atoms failed (either the occupation factor dropped down to zero or the displacement parameter increased to a physically unrealistic value) suggesting the chemical composition CuLiSn which contradicts the expectations from syntheses. A centrosymmetric structure model for CuLiSn exhibits an unusual fourfold coordination polyhedron around the atoms Sn and Cu/Li which is not in agreement with crystal chemical expectations. Also the volume per atom is too large for a formula CuLiSn (20.8 Å^³^) whereas it is in the expected range for CuLi_2_Sn (15.6 Å^³^). However, the *R* values are practically the same (*R*_1_=0.014 and *wR*_2_=0.038 for 46 reflections and 6 variable parameters; chemical formula CuLiSn; space group Fm-3m) as compared with the refinements in space group F-43m. (*R*_1_=0.013 and *wR*_2_=0.036 for 78 reflections and 9 variable parameters; chemical composition CuLi_2_Sn). Finally, the structural data for CuLi_2_Sn are given in the acentric space group due to crystal chemical reasons. The larger displacement parameter observed for the Cu/Li site (0.034 Å^²^) is to be mentioned; it might be an artefact of some systematic errors in the data set and high correlation terms. Refinements without a partial substitution of Cu at the site 4(*d*) increase the *R* value and the refinement tends not being stable.

The crystal structure of the CuLi_2_Sn phase is similar to the γ-Cu_3_Sn [13], [30] phase in the Cu–Sn binary system, which is BiF_3_-isotype. In γ-Cu_3_Sn the Sn and Cu atoms form a NaCl lattice and all cubic holes are filled consequently by further Cu atoms. Each interstitial Cu atom is quasi surrounded by four further Cu atoms and four Sn atoms. The cubic holes can be considered as a two-tetrahedral coordination of Cu by Cu and Sn, respectively. In CuLi_2_Sn, in contrast, Sn and Li atoms form the NaCl-lattice, interstitial cubic positions are split into 4(*c*)-(1/4 1/4 1/4) occupied solely by Cu atoms and 4(*d*)-(3/4 3/4 3/4) mainly occupied with Li atoms. Here each interstitial Cu or Li atom is quasi surrounded by four Li atoms and four Sn atoms. Analogue to γ-Cu_3_Sn, the cubic holes can be considered as a two-tetrahedral coordination of Cu or Li by Li and Sn, respectively. Tetrahedral fillings with same chemical environment are located on furthermost positions, corresponding to a distance of 4.4512(6) Å (half-length of face diagonal). In such an arrangement distances between atoms of the same kind are larger than in γ-Cu_3_Sn [30] (4.3258(6) Å). This effect is accompanied by the expansion of the lattice compared to γ-Cu_3_Sn.

The refined crystal structure is closely related to the ferromagnetic Heusler phases [31], [32] (space group Fm-3m, AlCu_2_Mn type), which have the general formula XY_2_Z. X is a main group element (group 13–15) at the 4(*a*) site 000 (e.g. Al), Y is a transition element at the 8(*c*) site (1/4 1/4 1/4) (e.g. Cu), and Z is a transition element at the 4(*b*) site (1/2 1/2 1/2) (e.g. Mn). This means that Al and Mn atoms form a NaCl lattice and all cubic holes are filled with atoms of one kind, namely Cu. In the CuLi_2_Sn structure, as already mentioned, the NaCl-like lattice is formed of Sn atoms, which is a group 13–15 element, and from Li, which is an alkali element. Furthermore the cubic holes are not filled solely by Cu atoms, but by Cu and by positions mixed occupied by both Li and Cu atoms. These two differences to the Heusler phase result in a non-ferromagnetic, but as reported weak diamagnetic behaviour [19].

**Fig. 4:**
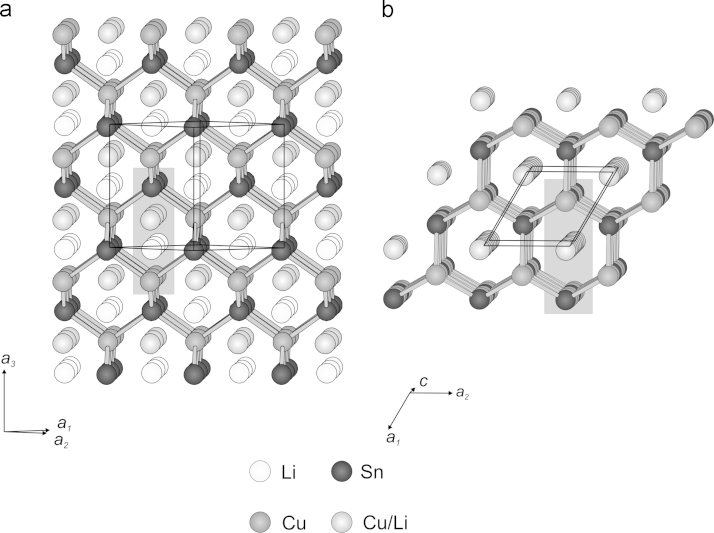
Comparison of channel structure of CuLi_2_Sn and Cu_2_LiSn.

**Fig. 5:**
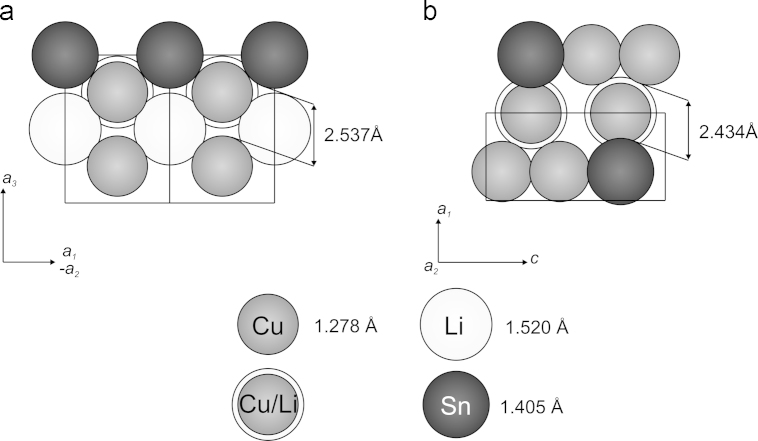
Comparison of vertical sections of channels in (a) CuLi_2_Sn and (b) Cu_2_LiSn according to shaded area in Fig. 4a and b; mixed Cu/Li-sites in Cu_2_LiSn are only shown for 2*(a)*- and 2*(b)*-positions.

Along the [110] direction (face diagonal), chair-shaped CuSn-hexagons show up which are condensed via adjacent edges and show a honeycomb-like structure in the projection (see Fig. 4a). The corners of the chair-shaped hexagons consist of shifted stacks of Cu and Sn atoms, respectively. The shifting vector between atoms of the Cu and Sn stacks is 2.2256(3) Å, which is a quarter of the unit cell׳s face diagonal. The formed hexagonal CuSn channel contains two piles with Li and mixed Cu/Li atoms each, which are also shifted to each other by a shifting vector of 2.2256(3) Å. Fig. 5a shows the vertical section of such a channel according to the shaded area in Fig. 4a. The atoms could be considered as ideal spheres with purely atomic radii [33]. However, this only approximates the real relations in the crystal lattice, because polarization effects due to different electronegativity values between the Cu, Li and Sn atoms, respectively, would modify the atomic radii significantly (e.g. Li: *r*_atom_=1.520 Å, *r*_covalent_=1.230 Å, *r*_ionic_=0.780 Å, values taken from Emsley [33]). The open diameter of the Li-conducting channel is 2.537(4) Å, which would allow an atom with a radius of 1.268(2) Å to penetrate. This is somewhere between the atomic and the covalent radius of Li and raises hope that this material could be interesting for technical applications.

### 4.2 Cu_2_LiSn

**Fig. 6:**
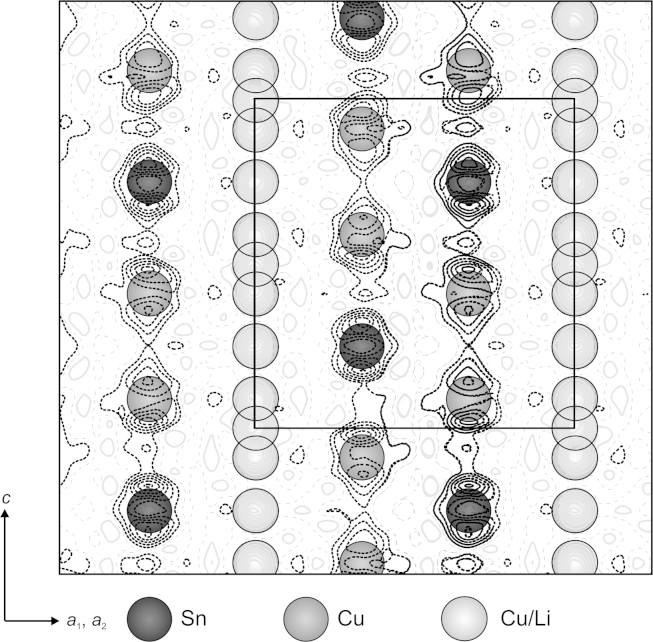
Cu_2_LiSn: Difference electron densities between measured and calculated pattern in [−110] direction, considering only 2(*c*)-Sn and 4(*f*)-Cu atoms in calculation. Negative, positive and no electron density differences are indicated with bold dashed, bold solid and normal dashed lines, respectively. Contour lines are graduated in steps of 0.5 e^−^/Å^3^. Excess electron clouds (solid lines) on 2(*a*)-, 2(*b*) and 4(*e*)-sites can be described by Cu/Li mixed occupations.

The refinement was started from the atomic coordinates given by Kripyakevich and Oleksiv [20]. In addition to the sites 2(*c*), 4(*f*) and 2(*a*) given by the former authors, the difference Fourier summation showed a significant electron density also at 4(*e*) and 2(*b*) (Fig. 6). Site 2(*c*) is occupied by Sn atoms and site 4(*f*) by Cu atoms.

**Fig. 7:**
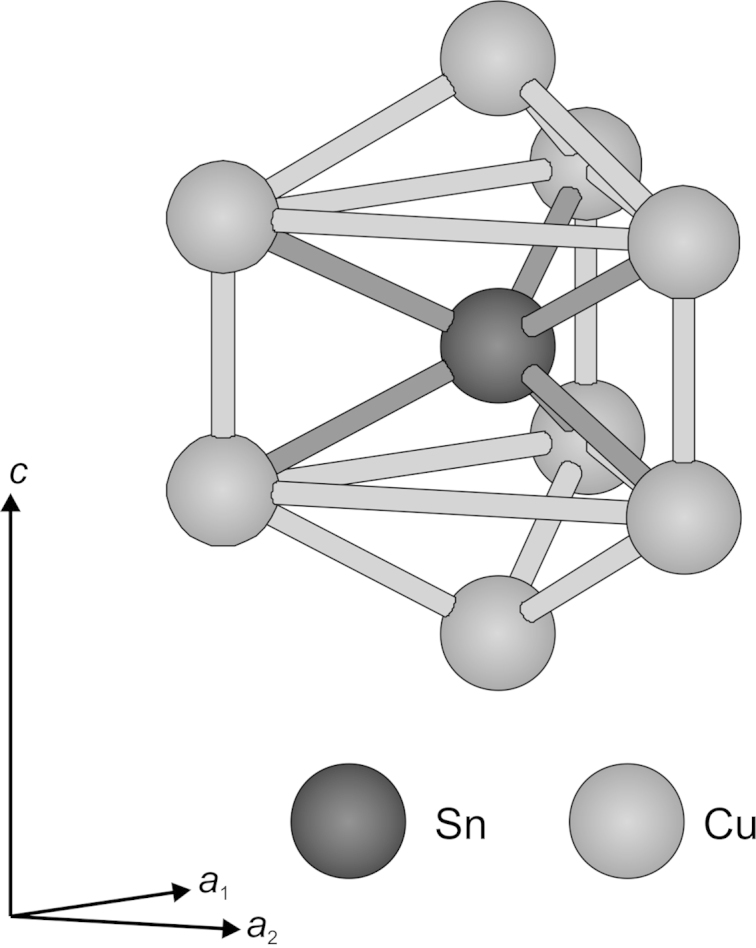
*Di*capped trigonal prismatic coordination polyhedron as backbone structure of Cu_2_LiSn, only 2(*c*)-Sn and 4(*f*)-Cu atoms are shown. Cu/Li atoms in channels are neglected in drawing.

The Cu atoms located at the 4(*f*) site form six-membered rings with chair configuration. Mirror inverted pairs of chair-shaped Cu rings are stacked in the [001] direction and centred in *z*=0.09/0.91 and *z*=0.41/0.59, respectively. The Sn atoms at 2(*c*) are located within the Cu chains forming the channels along [001]. Two Cu atoms are separated by one Sn atom. Considering its coordination sphere it is surrounded by eight Cu atoms forming a *di*capped trigonal prismatic coordination polyhedron (Fig. 7).

**Fig. 8:**
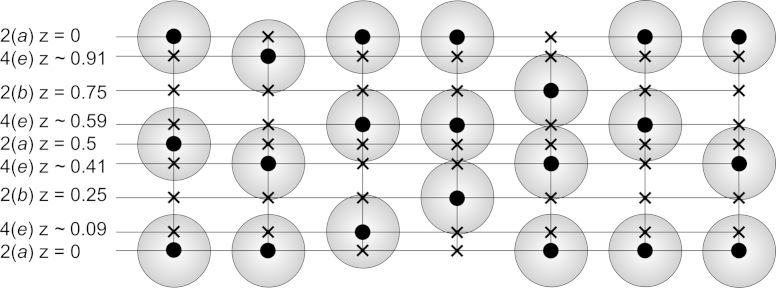
The seven possible models for the partially occupied (Cu/Li)-positions in the Cu_2_LiSn phase (possible (•) with corresponding impossible (*x*) positions, each vertical axis shows one channel along one unit cell).

The three additional sites labelled Cu/Li1 to Cu/Li3 require further discussion due to (1) too short contacts between these positions, (2) excess of electrons as compared to a full occupation of any of these three sites with Li atoms and (3) stoichiometry. In total there are eight atom positions for the Cu/Li atoms in the unit cell, all located at (00*z*). Full site occupation can be realised solely either on 2(*a*) or 2(*b*). An occupation of the site 4(*e*) only requires half occupation; ordering requires a reduction of space-group symmetry to *P*6_3_*mc*. If either site 2(*a*) or 2(*b*) is fully occupied, two atoms per unit cell are possible. If the sites 2(*a*), 2(*b*) and 4(*e*) are partially occupied, the maximum filling level per unit cell is 2.5 atoms to avoid too close atom–atom contacts. This can be achieved either by a combination of the partially occupied positions 2(*a*) and 4(*e*) or by 2(*a*), 2(*b*) and 4(*e*) (for possible combinations of partially occupied positions see Fig. 8). It could be assumed, that the mixed Cu/Li atoms at the 2(*b*), 4(*e*) and 2(*a*) sites are somehow mobile along the (00*z*) axis and migrate along channels (Fig. 4b), built up from the eightfold coordination polyhedron (Fig. 7).

Due to the mixed occupations of the 2(*b*), 4(*e*) and 2(*a*) sites, including the occupation ratios, the chemical formula in this case has two variable parameters: Cu_2+*x*_Li_1−*y*_Sn. Despite a full occupation of none of the three Cu/Li sites is possible considering all of them, the electron density exceeds the expected scattering power of two Li atoms per unit cell; the refinement converges for 4.8 Li atoms per unit cell. Consequently a mixed occupation is considered. Due to crystal chemical experiences, a partial substitution by Cu atoms seems most probable. Trials refining the structure model with variable Cu:Li ratios failed; therefore a refinement with the scattering power of Li alone and allowing an excess of atoms was performed (see Table 3, footnote a). The occupation of the sites 2(*a*), 2(*b*) and 4(*e*) is not completely statistical but exhibits some order mechanisms. It is verified by a significant elongation of the X-ray scattering reflections parallel to *c* (see Table 3, footnote b). In a few cases it was possible to resolve them as satellite reflections. However, most of the main and their satellite reflections are overlapping and a separate measurement was not possible; a **q** vector could not at all be determined. Anyhow, the crystal structure of this phase has to be considered as incommensurate with an extended portion of disorder.

The before mentioned channels in this phase are, similar to the CuLi_2_Sn phase, probably wide enough to allow a migration of Li and Cu atoms, respectively. Fig. 5b shows a vertical section of the channels according to the shaded area in Fig. 4b. The open diameter of the Li-conducting channels is 2.434(5) Å. This is less than in the CuLi_2_Sn phase, but in the Cu_2_LiSn phase the channels are filled only with one pile of Cu/Li atoms. This leads to shorter diffusion paths along the channel, what could be beneficially for the Li-“ion” migration.

**Fig. 9:**
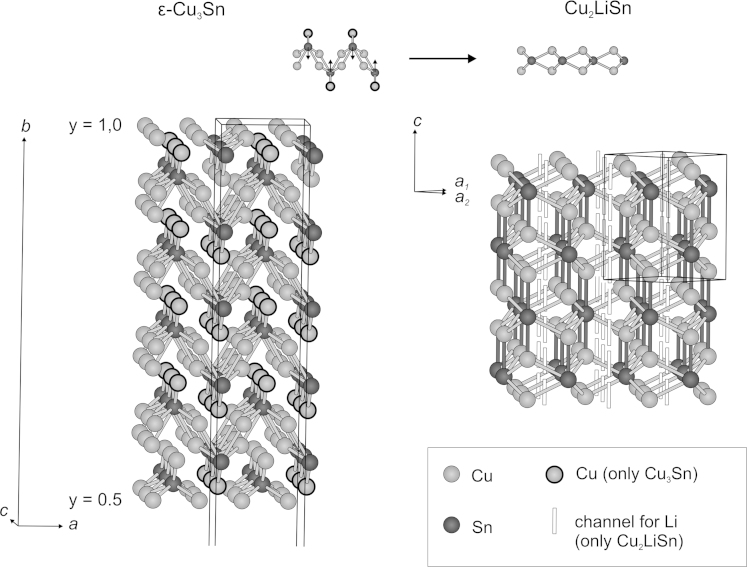
Relations between ε-Cu_3_Sn and Cu_2_LiSn. Shorter Cu–Sn bonds in Cu_2_LiSn are shown in dark grey.

The structural relationship between the binary phase ε-Cu_3_Sn [34] and the corresponding Cu_2_LiSn phase are not obvious on the first glance, but astonishingly close. Considering the half unit cell of the ε-Cu_3_Sn phase, it consists of parallel zigzag layers of Cu_2_Sn subunits. A third Cu atom that corresponds to each Sn atom is opposed to the Cu atoms belonging to the Cu_2_Sn subunits, forming a ridge along the Sn atoms (see Fig. 9). By aligning the Sn atoms into the centre of the Cu_2_Sn subunits and removing the opposing Cu atom on the ridge, the hexagonal backbone of the Cu_2_LiSn phase is formed. The Cu atoms of one layer are now located in a shorter distance to the closest Sn atom of another layer (*d*_Cu−Sn_=2.5825(6) Å) than to that one within the same layer (*d*_Cu−Sn_=2.7702(7) Å). During this alignment of the Cu_2_Sn layers the previous mentioned honeycomb-shaped (Cu/Li)-channels open up and Li atoms may be inserted (compare Fig. 4b).

## 5 Conclusions

The compounds CuLi_2_Sn and Cu_2_LiSn have been re-investigated by powder and single-crystal diffractometry. For CuLi_2_Sn and Cu_2_LiSn ordered CuSn and Cu_2_Sn lattices, respectively, were found. Extensive Cu–Li substitutions were proofed from structure refinements. This is in accordance with the variable chemical composition and the extension in both phase fields towards higher Cu concentrations at constant Sn content. Both structures could be relevant for the application of Cu–Sn alloys as anodes in Li-ion batteries. The theoretical capacity of CuLi_2_Sn is 273 mAh/g, that one of Cu_2_LiSn is 106 mAh/g. Besides beneficial gravimetric capacities also sufficient kinetic properties are mandatory. Several studies have investigated the electrochemical performance of the CuLi_2_Sn phase [6], [8], [35], [36], one in particular which measured the kinetic properties [37]. On the other hand, the kinetic properties of Cu_2_LiSn have not been treated by literature and are therefore still unknown. These would be of great interest hence the Cu–Sn main body of Cu_2_LiSn shows very close structural relationship to ε-Cu_3_Sn. A direct equilibrium of both phases caused by lithiation can be assumed. Generally, the mechanism of Li insertion and the reaction schemes are still widely unknown. The elucidation of these questions will be substantial for further works. Further details on the structure refinement are available from FIZ Karlsruhe [38] by quoting the registry numbers CSD-427095 (CuLi_2_Sn) and CSD-427096 (Cu_2_LiSn).
